# Peer substance use overestimation among French university students: a cross-sectional survey

**DOI:** 10.1186/1471-2458-10-169

**Published:** 2010-03-29

**Authors:** Lionel Riou Franca, Bertrand Dautzenberg, Bruno Falissard, Michel Reynaud

**Affiliations:** 1 INSERM U669 - Maison de Solenn - 97, bvd de Port-Royal - 75679 Paris Cedex 14, France; 2 Université Paris 6 - Pierre et Marie Curie - 4 place Jussieu - 75005 Paris, France; 3 Faculté de Médecine Pierre et Marie Curie, UPRES EA2397 - 91, 105 boulevard de l'Hôpital - 75013 Paris, France; 4 Assistance Publique-Hôpitaux de Paris (AP-HP), Service de pneumologie et réanimation - GH Pitié Salpêtrière Division Montyon - 75651 Paris cedex 13, France; 5 Université Paris-Sud 11 and Université Paris Descartes 5 - UMR-S0669 - Maison de Solenn - 97, boulevard de Port-Royal - 75679 Paris cedex 14, France; 6 Assistance Publique-Hôpitaux de Paris (AP-HP), Hôpital Paul Brousse, département de santé publique - 12, avenue Paul-Vaillant-Couturier - 94804 Villejuif Cedex; 7 Assistance Publique-Hôpitaux de Paris (AP-HP), Hôpital Paul Brousse, unité fonctionnelle d'addictologie - 12, avenue Paul-Vaillant-Couturier - 94804 Villejuif Cedex

## Abstract

**Background:**

Normative misperceptions have been widely documented for alcohol use among U.S. college students. There is less research on other substances or European cultural contexts. This study explores which factors are associated with alcohol, tobacco and cannabis use misperceptions among French college students, focusing on substance use.

**Methods:**

12 classes of second-year college students (n = 731) in sociology, medicine, nursing or foreign language estimated the proportion of tobacco, cannabis, alcohol use and heavy episodic drinking among their peers and reported their own use.

**Results:**

Peer substance use overestimation frequency was 84% for tobacco, 55% for cannabis, 37% for alcohol and 56% for heavy episodic drinking. Cannabis users (p = 0.006), alcohol (p = 0.003) and heavy episodic drinkers (p = 0.002), are more likely to overestimate the prevalence of use of these consumptions. Tobacco users are less likely to overestimate peer prevalence of smoking (p = 0.044). Women are more likely to overestimate tobacco (p < 0.001) and heavy episodic drinking (p = 0.007) prevalence. Students having already completed another substance use questionnaire were more likely to overestimate alcohol use prevalence (p = 0.012). Students exposed to cannabis prevention campaigns were more likely to overestimate cannabis (p = 0.018) and tobacco use (p = 0.022) prevalence. Other identified factors are class-level use prevalences and academic discipline.

**Conclusions:**

Local interventions that focus on creating realistic perceptions of substance use prevalence could be considered for cannabis and alcohol prevention in French campuses.

## Background

Social norms interventions have been proposed in order to reduce substance use in higher education. These interventions are based on the social norms theory, which claims that students overestimate the prevalence and acceptability of substance use in campus, and will align their behaviours with these beliefs [1]. Different psychological mechanisms have been described to explain why students will overestimate the norms of substance use. They include false consensus, according to which the individuals engaging in unhealthy behaviours will think their patterns of use are the norm, pluralistic ignorance, according to which the majority of individuals with healthy behaviours will falsely think they are not behaving accordingly with the norm, and of false uniqueness, according to which the individuals who do not engage in problematic behaviours will falsely think they are more unique than they really are [2]. Substance use misperceptions are therefore associated with individual use.

There are several ways to conduct a social norms prevention intervention. It can be tailored in the form of a social norms marketing campaign, in which accurate, health promoting norms are delivered community wide. These campaigns have been effective in reducing student drinking [3], although the findings have not always been replicated [4]. Targeted social norms interventions, on the opposite, focus only on members of a particular group. Positive results have also been reported [5-8]. Finally, individualized social norms interventions target high risk substance users or abusers, as part of individual counselling interventions, and have also been encouraging in reducing alcohol use [9-11].

Before engaging in a social norms prevention intervention, it is necessary to assess what misperceptions exist with respect to the behaviour to target and if these misperceptions are associated with the behaviour.

There is a growing volume of literature showing that, for the substances most used by higher education students (tobacco, alcohol and cannabis) [12-15], misperceptions do exist [16]. Literature reviews [17,18] and meta-analyses [19] conclude that misperceptions of alcohol use among college students do exist and that they are partially correlated with increased personal consumption.

Most studies focusing on tobacco have been performed in young teenagers. Some studies indicate that perceived smoking prevalence is overestimated particularly by smokers [20-24], although this finding is inconsistent: in some multivariate models, perceived prevalence of smoking has been reported to be inversely associated with smoking [25].

Less research exists on cannabis, but misperceptions of the norms and an association with individual use have also been reported among higher education students [16,26-28].

Other factors can also influence misperceptions. Women have been shown to be more prone to misperceptions than men, at least for alcohol use [19,29]. While the social norms theory focuses on the link between the perception of the use of a given substance and its use, misperceptions of one particular substance can be associated with the use of another [30]. Furthermore, for tobacco use, noticing other peers smoking (i.e. visibility of the behaviour) is associated with smoking among teenagers [25] and could also influence the perception of the norms. Berkowitz questions the impact of other prevention campaigns: "When drug prevention emphasizes problem behavior without acknowledging the actual healthy norm, it may foster the erroneous belief that drinking problems are worse than is actually the case and inadvertently contribute to the problem it is trying to solve" [2]. Finally, as substance use has been shown to vary from one academic discipline to another [31,32], specific discipline sub-cultures could influence misperceptions.

Various degrees of evidence in favour of the existence of substance use misperceptions among higher education students and of an association between these misperceptions and substance use are therefore available. Most published research, however, focuses on U.S. higher education students. The question of the generalisability of these results to other cultural settings remains. For instance, in France, alcohol consumption begins at a younger age (the legal drinking age is 16 years), cannabis use rates are among the highest in Europe while heavy episodic drinking is less frequent [33], and few students are accommodated in student residences. All these factors could influence both the perception of the norms of substance use and the degree of influence of peer students as compared to other social groups relevant to the students.

The purpose of this analysis is to assess if misperceptions of tobacco, cannabis and alcohol use exist among French higher education students, and which factors influence these misperceptions. Should the U.S. literature findings be directly applicable to the French setting, we expect students engaging in substance use to be more prone to overestimation than non users, adjusting for other predictors. Moreover, to our knowledge, prevention campaigns based on the social norms theory have never been implemented in France. This fact allows for the exploration of the impact of prevention approaches based on other rationales on the perceptions of substance use.

## Methods

### Participants

The REACTIF study, conducted among higher education students from Île-de-France, included a self-reported questionnaire to measure alcohol, cannabis and tobacco consumption in addition to individual norms regarding their use. The study was approved by the institutional review board of the French institute of health. Second year undergraduate students were surveyed between October 2005 and February 2006. A researcher personally administered an anonymous paper and pencil questionnaire to students, enabling study objectives to be presented and assistance in completing the questionnaire to be provided. Questionnaires were administered during a regularly scheduled lecture. Data capture was automatic, using an optical mark recognition system.

To reduce variability between sampling units, we restricted ourselves to Paris and the neighbouring Île-de-France region and to 4 specific academic disciplines. Furthermore, we tried to select all classes from each academic discipline in the same geographical unit: (1) Paris or (2) its region.

12 classes selected at random were surveyed during a lecture. All students present were asked to participate. Nursing school classes (n = 4) were selected among Parisian nursing schools. Sociology (n = 3) and applied foreign language (n = 3) classes were selected in the Paris region. Finally, two medicine classes, one in Paris and one in the Paris region (due to the lack of medicine universities in the Parisian region, we could not ensure homogeneity of the geographical units for this discipline), were selected.

The choice of the disciplines was based on their specificities of substance use: nursing students have a high prevalence of smoking in France [34], as opposed to medicine students. Sociology students have a high prevalence of cannabis use [32]. Applied foreign language is a three year undergraduate program which combines the study of two languages with economic disciplines. Students are then qualified to work in international trade. This discipline was chosen in order to include a group without higher than average substance use.

### Outcome measures

We have chosen a simple and widely used [25,35-37] descriptive social norms measure: the perceived prevalence of substance use. Although some authors question the utility of this measure for tobacco peer norms, as it performs inconsistently on predictive models when other types of peer influences are also measured [25], we have focused on this variable since these perceptions are easily modifiable by providing the students more accurate consumption prevalences, estimated directly from their own answers to the survey. Students were asked to estimate, among 10 peer students (students from the same academic setting), how many (1) smoke tobacco, (2) drink alcohol, (3) sometimes have 5 drinks or more in a row, (4) use cannabis (they were therefore asked to estimate usual behaviours, without any reference to a time frame of occurrence). Peer students are therefore students in the same field of studies and the same campus, but not necessarily in the same class (only those in their second year of studies were surveyed). We did not use an open-ended prevalence estimate in order to minimize missing values (as students showed some difficulties in estimating this value, it was easier for them to tick one value among 11 than to estimate it from scratch) and to facilitate data capture. Our variable of interest, however, is substance use overestimation. We therefore have to compare actual norms, as estimated from the data gathered among second-year students, to perceived norms, which were measured in a more general setting (peer students from the same academic setting). The choice of a larger basis for the estimation of perceived norms results from a trade-off between precision of the estimation of misperceptions and both utility and feasibility of the study. The results of the survey were to be used for later prevention interventions (this fact helped to motivate the universities to participate in the study) and we wished to reach for a larger audience when communicating the results than just the students from the surveyed class. Estimating perceived norms in a broader setting than only the class of second-year students allowed for this goal to be reached.

We therefore use actual norms among second-year students as an estimator of actual norms among all students from the same academic setting (which also includes first and third-year students from the same field of studies). For overestimation to occur, a student must provide an estimate of peer substance use greater than the prevalence measured within his class. The latter is measured with a precision of 10% to match the social norms measure. For example, for a class prevalence of 28%, if a student estimates that three students among 10 use a substance, there is no overestimation. If he provides an estimate of four students among 10 (or more), he is considered to be an overestimating student for the corresponding substance.

### Independent variables

In order to assess whether there was an association between substance use overestimation and the consumption levels, we defined three categories: "no use", "low use" (i.e. occasional) and "high use" (i.e. regular). We used past consumption instead of usual consumption to make results comparable with other studies [38,39]. The cutoffs used for all four consumptions were also chosen in order to match commonly used definitions, in particular those of the ESCAPAD study [39], who defines regular use for alcohol and cannabis as at least 10 episodes in the past month. For cannabis, the cuttof between "low" and "high" use was of more than one use per week in the past year. For tobacco, it was of use every day in the past month (i.e. daily use). Cutoff for "low" and "high" use of alcohol drinking was of 10 days in the last month. A cutoff of 4 episodes in the month (approximately once a week) was used to define "low" and "high" use of heavy episodic drinking (5 drinks or more on a single occasion).

In addition to substance use, the following other covariates were tested as predictors of substance use overestimation: gender, age, discomfort with smoking in the university as a measure of tobacco use visibility in campus, attitude concerning smoke-free universities, number of tobacco, alcohol or cannabis prevention campaigns seen in the past month, previous experience with substance use questionnaires, prevalence of substance use within their class and academic discipline as measures of class culture.

### Statistical analysis

Analyses were performed using the R Statistical programming language [40].

In order to respond to the main objective of this study, to validate the hypothesis that substance users are more likely to overestimate peer substance use, four regression models (for tobacco, cannabis, alcohol use and heavy episodic drinking) were fitted. The dependent variables were the overestimation of each substance use by the students. In order to select which variables to include in the regression model, a bootstrap selection procedure was used [41]. 1000 bootstrap samples of the data were generated and backward variable selection based on the Akaike's Information Criterion (AIC) was performed on each of the samples. All variables that were likely to be selected on the basis of the AIC were retained in the final model. The selection threshold was arbitrarily chosen at 55% (probability for the variable to be selected in a bootstrap sample) in order to include all potentially statistically significant variables. The significance of the association between the covariate and the dependent variable was assessed using the likelihood ratio test. P-values < 5% were considered significant.

Students from the same class are clustered and this grouping could induce a correlation structure in the error terms of the regression models. We explored this possibility using a random-effect model and testing the significance of the random effects using bootstrap procedures [42]. As no evidence of clustering was found (p = 0.44 for tobacco, 0.78 for cannabis, 0.68 for alcohol and 0.73 for heavy episodic drinking), we retained the standard logistic regression model for all analyses.

## Results

### Demographics and substance use

731 students were retained for the analysis. Table 1 presents the demographic characteristics of the sample.

**Table 1:** Demographic characteristics of the disciplines sampled

	**F. Lang**.	Medicine	Nursing School	Sociology	All
Class level variables					

Mean sample size (range)	29 (25 - 33)	68 (64 - 72)	62 (44 - 90)	87 (55 - 125)	61 (25 - 125)

% women (range)	82 (73 - 90)	78 (75 - 80)	87 (83 - 93)	70 (65 - 77)	79 (65 - 93)

Student level variables					

Mean age (range)	20.4 (18 - 27)	19.9 (18 - 24)	24.6 (18 - 48)	20.9 (18 - 64)	21.9 (18 - 64)

Median age (interquartile interval)	20 (19 - 20)	19 (19 - 20)	22 (20 - 25)	20 (19 - 21)	20 (19 - 21)

The population was mostly comprised of sociology students (36%) and nursing students (34%). 79% of students were women (gender is missing for 5 students). Students who have not repeated a year enter the second year of university at age 19-20. In nursing schools, however, many tend to have a professional or educational background before beginning their studies, and students are therefore older. Table 2 shows the prevalence of tobacco (1 missing value), cannabis (7 missing values), alcohol use (no missing values) and heavy episodic drinking (2 missing values) aggregated according to academic discipline and to gender.

**Table 2:** Prevalence of substance use, academic discipline and gender

Substance use (%)	Academic Discipline				Gender	
	**F. Lang. (n = 87)**	**Medicine (n = 136)**	**Nursing School (n = 248)**	**Sociology (n = 260)**	**Men (n = 156)**	**Women (n = 570)**

**Tobacco**				p < 0.001		p = 0.361

No use last month (64.9%)	78.2	77.9	59.9	58.5	61.5	65.7

Occasional use (19.6%)	13.8	17.6	22.7	19.6	19.2	19.7

Daily use last month (15.5%)	8.0	4.4	17.4	21.9	19.2	14.6

**Cannabis**				p < 0.001		p < 0.001

No use last year (70.7%)	83.9	71.6	75.4	61.4	52.6	75.8

≤ 1/week last year (22.0%)	12.6	34.6	21.3	24.3	31.8	19.1

> 1/week last year (7.3%)	3.4	3.7	3.3	14.3	15.6	5.1

**Alcohol**				p < 0.001		p < 0.001

No use last month (30.9%)	49.4	26.5	30.6	27.3	17.3	34.4

1-9 days last month (60.1%)	49.4	64.0	64.1	57.7	59.6	60.4

10+ days last month (9.0%)	1.1	9.6	5.2	15.0	23.1	5.3

**Heavy episodic drinking**				p < 0.001		p < 0.001

No use last month (71.5%)	89.5	65.4	71.8	68.3	51.0	77.0

1-3 times last month (21.7%)	9.3	27.9	25.0	19.3	34.2	18.3

4+ times last month (6.9%)	1.2	6.6	3.2	12.4	14.8	4.7

Note: p-values are for the Chi-square test of independence between substance use and academic discipline or gender

High prevalences of all four consumptions were observed among sociology students: 42% smoked tobacco, 73% drank alcohol, 32% report heavy episodic drinking episodes in the last month; 39% used cannabis in the last year. In contrast, applied foreign language students have the lowest consumption levels (respectively 22%, 50%, 10% and 16% for tobacco, alcohol, heavy episodic drinking and cannabis).

Nursing students along with sociology students have the highest prevalences of tobacco smoking (40%). As for medicine students, they have a low prevalence of tobacco use (22%), but have a prevalence of heavy episodic drinking (35%) similar to sociology students. There is, however, a difference: medicine students appear to have more occasional uses of cannabis and alcohol whereas sociology students are more frequently regular users of these substances. 13% of medicine students reporting having used cannabis in the last year used it more than once per week, as opposed to 37% of sociology students; the figures are of 19% (medicine) and 39% (sociology) for the proportion of heavy episodic drinkers reporting four or more episodes of heavy episodic drinking in the last month. Except for tobacco, men are more frequently substance users than women.

### Perceived substance use

**Figure 1:**
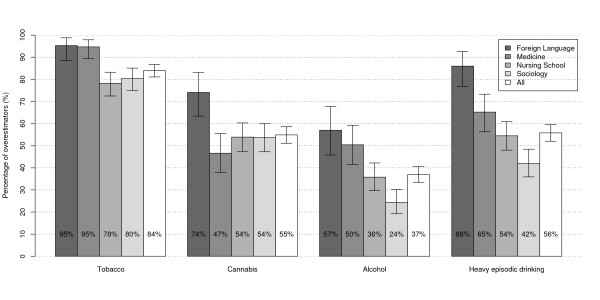
**Prevalence of substance use overestimation by academic discipline and 95% confidence intervals**. p ≤ 0.001 for the chi-squared test of independence between academic discipline and substance use overestimation for all four behaviours.

Figure 1 shows the proportion of students who overestimate substance use. Most students (84%) overestimate tobacco use prevalence. Many students overestimate cannabis use prevalence (55%) and heavy episodic drinking (56%) whereas alcohol use prevalence often seems to be correctly perceived (37%). There is an association between academic discipline and substance use overestimation (p ≤ 0.001 for all four substances), and applied foreign language students are the most likely to overestimate.

### Predictors of substance use overestimation

Table 3 presents the variables that were selected (probability of inclusion in the bootstrap selection procedure of at least 55%) and their significance in each of the four logistic regression models (likelihood ratio test).

**Table 3:** Statistical significance of the variables used in the models predicting substance use overestimation

Variable	p-value (Likelihood Ratio Test)			
	**Tobacco**	**Cannabis**	**Alcohol**	**HED**

Tobacco use	**0.0438**	-	0.0997	**0.0059**

Cannabis use	-	**0.0062**	-	-

Alcohol use	-	0.0632	**0.0029**	-

HED	-	-	-	**0.0019**

Discipline	-	**0.0007**	**<0.0001**	**<0.0001**

Tobacco use prevalence in class	**<0.0001**	-	-	-

Cannabis use prevalence in class	-	**0.0004**	-	-

Alcohol use prevalence in class	-	0.0657	**0.0055**	**0.0042**

HED prevalence in class	-	-	**0.0267**	**<0.0001**

Gender	**0.0002**	0.0569	-	**0.0069**

Previous questionnaires	-	-	**0.0122**	0.0893

Cannabis prevention campaigns	**0.0217**	**0.0177**	-	-

A "-" sign indicates that the variable was not included in this model. Bold p-values indicate significance at the 5% level. HED: Heavy episodic drinking.

Tobacco use overestimation is associated with tobacco use, prevalence of smoking in class, gender and the number of cannabis campaigns seen in the last month.

Table 4 gives the adjusted odds ratios (ORs) and associated 95% confidence intervals (CIs) estimated from the four logistic regression models for tobacco, cannabis, alcohol and heavy episodic drinking use.

**Table 4:** Adjusted ORs and Confidence intervals for substance use overestimation

Variable	Adjusted OR (95% confidence interval)			
	**Tobacco**	**Cannabis**	**Alcohol**	**HED**

Tobacco use (reference: no use)				

Low: < 1/day in the last month	0.68 (0.40 - 1.17)	-	NS	0.64 (0.41 - 0.99)

High: ≥ 1/day in the last month	0.52 (0.30 - 0.89)			0.47 (0.28 - 0.77)

Cannabis use (reference: no use)				

Low: ≤ 1/week in the last 12 months	-	1.66 (1.11 - 2.51)	-	-

High: > 1/week in the last 12 months		2.37 (1.23 - 4.73)		

Alcohol use (reference: no use)				

Low: < 10 days in the last month	-	NS	1.95 (1.31 - 2.92)	-

High: ≥ 10 days in the last month			2.17 (1.10 - 4.23)	

HED (reference: no use)				

Low: < 4 times in the last month	-	-	-	1.85 (1.19 - 2.89)

High: ≥ 4 times in the last month				2.64 (1.35 - 5.29)

Discipline (reference: sociology)				

Foreign language	-	0.78 (0.32 - 1.93)	0.44 (0.13 - 1.43)	0.75 (0.21 - 2.68)

Medicine		0.34 (0.18 - 0.64)	3.72 (2.28 - 6.11)	3.46 (2.12 - 5.70)

Nursing studies		0.35 (0.18 - 0.70)	1.16 (0.72 - 1.85)	1.01 (0.66 - 1.54)

Tobacco use prevalence in class	0.92 (0.89 - 0.94)	-	-	-

Cannabis use prevalence in class	-	0.92 (0.88 - 0.96)	-	-

Alcohol use prevalence in class	-	NS	0.96 (0.93 - 0.99)	1.04 (1.01 - 1.07)

HED prevalence in class	-	-	0.93 (0.87 - 0.99)	0.85 (0.79 - 0.91)

Gender (reference: male)	2.51 (1.56 - 4.02)	NS	-	1.75 (1.17 - 2.64)

Previous questionnaires (reference: no)	-	-	1.56 (1.10 - 2.21)	NS

Cannabis prevention campaigns	1.15 (1.02 - 1.34)	1.11 (1.02 - 1.22)	-	-

A "-" sign indicates that the variable was not included in this model. NS: the variable was included in the model but failed to reach significance at the 5% level. HED: Heavy episodic drinking.

Tobacco users are less likely to overestimate smoking prevalence, and women are more likely to overestimate it. The higher the prevalence of smoking in class, the less likely it is for a student to be an overestimator. The probability of overestimation increases with the number of cannabis prevention campaigns seen in the last month.

Cannabis use overestimation is associated with academic discipline, cannabis use, cannabis use prevalence in class, and the number of cannabis prevention campaigns seen in the last month. The probability of overestimation is increased for cannabis users and for sociology students. It also increases with the number of cannabis prevention campaigns seen in the last month. It decreases with the prevalence of cannabis use in class.

Alcohol use overestimation is associated with academic discipline, alcohol use, alcohol and heavy episodic drinking prevalence in class, and previous exposure to substance use questionnaires. The probability of alcohol use overestimation is increased for alcohol users, medicine students, and students having already completed substance use questionnaires in the past. It decreases with the prevalence of alcohol and heavy episodic drinking in class.

Heavy episodic drinking overestimation is associated with academic discipline, gender, heavy episodic drinking, tobacco smoking, alcohol and heavy episodic drinking prevalence in class. The probability of overestimation of heavy episodic drinking is increased for heavy episodic drinkers, women, medicine students and non smokers. It decreases with the prevalence of heavy episodic use in class, but increases with the prevalence of alcohol use.

## Discussion

Our study finds evidence for substance use misperceptions among French higher education students. Only 37% overestimate class alcohol use prevalence, since this prevalence is high (69% of the students), but 84%, 56% and 55% of the students respectively overestimate class tobacco smoking, heavy episodic drinking and cannabis use prevalence. We also find evidence of an association with substance use. Thus, social norms prevention campaigns should be appropriate in these contexts. We will now review the principal factors identified as associated with misperceptions in our study.

### Substance use and overestimation of the norms

Cannabis users, alcohol and heavy episodic drinkers are more at risk of overestimating the respective peer substance use norms. However, these users are not more at risk of overestimating class prevalence of other substance use. These associations between substance use and the misperception of its use among class peers are in agreement with other studies focusing on alcohol [17] and cannabis [26,27]. One possible explanation for this association can be derived from the concept of self-serving bias: heavy substance users have a personal motivation for overestimating the norms, since it allows them to justify their own use and deny it is problematic [2]. The association can however also be explained by the fact that the students that are more prone to overestimating group norms are more likely to increase their own substance use in order to meet the perceived expectations, as postulated by the social norms theory [2].

Tobacco use represents an exception. Tobacco users are *less *likely to overestimate both tobacco use and heavy episodic drinking norms. Comparisons with other studies are limited by the fact that they do not apply to the same populations. Tobacco smoking research mostly focuses on teenagers whereas our results relate to 20-year old students. In a longitudinal study among students from 10 to 15 years of age, the authors failed to find an association between perceived peer cigarette use at baseline and personal cigarette use at the end of the study [30]. Some other studies report that smokers are more likely to overestimate smoking prevalence [20-22] while others report the contrary [25]. The link between tobacco use and social norms appears therefore to vary greatly from one setting to another.

This analysis focused on perceived prevalence of substance use among peer students, as it is the easiest norm to target for prevention purposes. Other types of norms have also been measured in this study and assessed elsewhere [43,44]. For tobacco use, other norms appear to be more strongly linked to smoking [43].

### Gender and substance use misperceptions

Female students more frequently overestimate tobacco smoking and heavy episodic drinking prevalences. This pattern has been frequently noticed in social norms studies [2,19,29] and different hypotheses have been proposed to explain it. Women could be more susceptible to environmental influences, or may be less involved in the cultures of substance use and misperceive them more [2]. Drinking behaviours differ between US and French higher education students: in our survey (end 2005), 83% of males and 66% of females were alcohol drinkers; 49% and 23% were heavy episodic drinkers (see table 2). The Harvard school of public health college alcohol survey was one of the largest studies of alcohol use in a nationally representative sample of U.S. college students. According to this survey, in 2001, 80% of U.S. male students and 82% of females were not abstainers; 49% of males and 41% of females were heavy episodic drinkers [45]. Another representative source of data arises from the Monitoring the Future study, with data available until 2007 for college students. In 2005, 71% (67% in 2007) of males and 66% (66% in 2007) of females had used alcohol in the last 30 days; the figures were of 49% (50% in 2007) and 36% (34% in 2007) for students having engaged in heavy episodic drinking in the past two weeks [46,47]. Thus, for alcohol, gender gaps appear to be more pronounced in our study, which is coherent with the previous hypotheses. However, we find no gender gap for tobacco smoking (p = 0.36 in a bivariate analysis).

Another explanation for the fact that women are more prone to overestimation may be due to the fact that students tend to consider a typical student as a male, and males are thought to be more frequent substance users than females [48].

### Class culture

Academic discipline is associated with overestimation of cannabis, alcohol and heavy episodic drinking norms. Furthermore, for all four norms studied, its prevalence of use at the class level was a significant predictor of overestimation. Class level factors are therefore likely to have an impact on both substance use and on the perception of its norms.

These results confirm the need for preliminary research before engaging in prevention interventions at the university level. They should help identify which substances are more problematic, and which norms are more misperceived.

For example, interventions targeting sociology students could focus on cannabis, as its use is both more prevalent and more misperceived among these students, while alcohol abuse prevention campaigns appear to be suitable for medicine students.

The tailoring of a social norms intervention on a particular group, however, should also take into account the issues of salience and relevance. The more a student identifies with his group of students, the more likely the correction of misperceptions among that group is to be effective [49]. Although this study assessed what proportion of the student's friends where also in the same class (median proportion of 37.5%, average proportion of 42.2%), it did not explore group identity.

### External influences

Students who report having seen many cannabis campaigns in the past month are more at risk of overestimating cannabis use (and also tobacco use) and students already exposed to substance use surveys are more at risk of overestimating alcohol use norms. These associations are adjusted for individual cannabis, tobacco or alcohol use. Our survey does not provide information about the type of cannabis campaigns seen or of substance use questionnaires previously completed. No national campaign based on social norms has been launched in France (the first cannabis-related national prevention campaign, launched in France at the period of the study, used "cannabis is a reality" as a catchphrase). It could be that media exposure on cannabis use could increase students' estimations of its prevalence, as exposure to alcohol-related questionnaires, without a feedback on the results, could lead to an increase on the perceived prevalence of alcohol use. The association between cannabis campaigns and tobacco use could be explained by the fact that cannabis use is known to be associated in France with tobacco use (as it is smoked in joints). The association between overestimation of the norms of use of the other substances and overestimation of the norms of use of a given substance was not tested in this analysis. The possible impact of prevention campaigns on perception of the norms of use has already been raised by Berkowitz, in a speculative fashion [2].

In surveys carried out among French high school students, it has been noted that substance users are more likely to report that "something has been done in the high school to prevent students from" smoking, drinking alcohol or using cannabis [50]. Moreover, drug prevention programs among children have been documented to have the potential to have a negative impact on drug use itself, particularly those using 'scare' tactics [51]. The existence of a link between drug education and drug use has therefore already been explored in the past, for a younger population. However, our study adds that, among higher education students, prevention campaigns and exposure to drug use questionnaires can be associated with perceived norms of use, independently of the students' level of use of the substances.

### Limitations

When interpreting the results of this study, some particularities of the design must be taken into account.

First, the associations reported in this study are based on a cross-sectional sample. Consequently, we don't know whether the independent variables occur before or after the dependent variables in the models (overestimation of substance use norms). Only an experimental study design to make inferences for causal effects.

Furthermore, the number of students enrolled in each class was not available. Although the participation rate of the students present during the administration of the questionnaire were very high, there is the possibility that a significant number of students were absent from class. When asked informally for this possibility, both students and teachers present during the administration declared that the number of students present was no different than usual. Medicine students in France have however higher absenteeism rates than other students. More frequently absent students might be more at risk of substance use, and therefore the prevalence of substance use among enrolled students might be underestimated when calculated on present students only. As social norms prevention interventions are necessarily based on present students, however, norms of use among usually present students might be of more utility than norms of use among both usually and rarely present students.

In order to judge the representativeness of the sample, we can compare the characteristics of the students participating in the study with overall statistics, in particular for gender. In France, as in most European countries, females are more represented than males in higher education [52]. The sex-ratio varies according to the disciplines studied, the percentage of women is of about 88% in nursing studies [53], 75% in foreign language, 70% in sociology and 66% in medicine (ministry of education data for 2004). Therefore, except for medicine students (females might be less likely to be absent from class), there are no particular differences in our sample (see table 1).

Finally, there is a possibility that part of the peer substance use overestimations measured in this study is due to methodological issues. Heavier consumers might have not been present when the study was administered. Students might have under-reported their own substance use, as this measure was based on self-reporting. While students were asked in the end of the questionnaire whether they felt they had always been honest when answering the questionnaire (three students giving a negative answer where excluded from analysis), measures of social desirability were not included in the questionnaire. Self-reported substance use questionnaires have, however, been shown to be reliable for the substances studied [54]. The issue of the group chosen to assess social norms can also be discussed. The estimate of social norms will be impacted by the students' perception of the reference group.

More importantly, in order to estimate misperceptions of peer student substance use, we used one specific class (second-year students) to estimate actual norms among the overall field of studies. Students were asked to provide descriptive norms about students from the same educational setting (same university and same field of studies), while actual norms of use are based only on students from the same class.

If these two populations are not comparable, the estimation of misperceptions will be biased and the validity of the estimate can be questioned. Are second-year students from a specific field of studies in a given university representative of all students from this field of studies and this university? If not, is the bias induced big enough to invalidate the conclusions? Substance use prevalences can differ in different years of studies, but these differences are likely to be negligible when compared with those arising from heterogeneity across different academic disciplines. For example, using data from a previous survey carried out among students from the Parisian region (the unpublished "Facultés et Ecoles Sans Tabac en Ile-de-France" survey), considering nursing students (to maximise the prevalences), the largest differences in smoking prevalence where between second-year students (46% smoke tobacco) and third-year students (40% smoke tobacco). This difference, of 6 percentage points, is below the threshold (of 10 percentage points) used to define a student as an overestimator. While we lack data for all academic disciplines and all substance use behaviours studied, we can therefore reasonably believe that the differences between actual substance use norms according to the year of studies are likely to be small enough for our estimate of overestimations to remain valid. The validity of the conclusions, however, depend on the plausibility of this hypothesis and this issue can be seen as the more salient limitation of this work.

## Conclusions

These results show that there are grounds for university level prevention campaigns based on local survey results. One first point is that substance use patterns and perceptions of the norms differ significantly across academic disciplines. A second point, according to our findings for cannabis, alcohol use and heavy episodic drinking, is that substance users are more likely to misjudge real peer use prevalence. Social norms of substance use are an important factor among students personal use. Overestimating these norms is associated with increased levels of use. In addition to other strategies, prevention programs should consider changing use perception when it is overestimated.

These results are original in that they do not focus on a single substance, but on the three main consumptions at the university level: tobacco, cannabis and alcohol, including heavy episodic drinking.

## Competing interests

The authors declare that they have no competing interests.

## Authors' contributions

LRF, BD and MR designed the study and wrote the protocol. LRF and MR undertook the pilot study. LRF and BD participated in the collection of the data. BF validated the statistical analysis plan. LRF managed the literature searches and summaries of previous related work, undertook the statistical analysis, and wrote the first draft of the manuscript. All authors contributed to and have approved the final manuscript.

## Pre-publication history

The pre-publication history for this paper can be accessed here:

http://www.biomedcentral.com/1471-2458/10/169/prepub
